# Inflammation in Sickle Cell Disease: Differential and Down-Expressed Plasma Levels of Annexin A1 Protein

**DOI:** 10.1371/journal.pone.0165833

**Published:** 2016-11-01

**Authors:** Lidiane S. Torres, Jéssika V. Okumura, Danilo G. H. Silva, Kallyne K. O. Mimura, Édis Belini-Júnior, Renan G. Oliveira, Clarisse L. C. Lobo, Sonia M. Oliani, Claudia R. Bonini-Domingos

**Affiliations:** 1 Laboratory of Hemoglobin and Hematologic Genetic Diseases, Department of Biology, São Paulo State University (UNESP), São José do Rio Preto, São Paulo, Brazil; 2 Laboratory of Immunomorphology, Department of Biology, São Paulo State University (UNESP), São José do Rio Preto, São Paulo, Brazil; 3 Institute of Hematology *Arthur de Siqueira Cavalcanti* (HEMORIO), Rio de Janeiro, Rio de Janeiro, Brazil; Université Claude Bernard Lyon 1, FRANCE

## Abstract

Sickle cell disease (SCD) is an inherited hemolytic anemia whose pathophysiology is driven by polymerization of the hemoglobin S (Hb S), leading to hemolysis and vaso-occlusive events. Inflammation is a fundamental component in these processes and a continuous inflammatory stimulus can lead to tissue damages. Thus, pro-resolving pathways emerge in order to restore the homeostasis. For example there is the annexin A1 (ANXA1), an endogenous anti-inflammatory protein involved in reducing neutrophil-endothelial interactions, accelerating neutrophil apoptosis and stimulating macrophage efferocytosis. We investigated the expression of ANXA1 in plasma of SCD patients and its relation with anemic, hemolytic and inflammatory parameters of the disease. Three SCD genotypes were considered: the homozygous inheritance for Hb S (Hb SS) and the association between Hb S and the hemoglobin variants D-Punjab (Hb SD) and C (Hb SC). ANXA1 and proinflammatory cytokines were quantified by ELISA in plasma of SCD patients and control individuals without hemoglobinopathies. Hematological and biochemical parameters were analyzed by flow cytometry and spectrophotometer. The plasma levels of ANXA1 were about three-fold lesser in SCD patients compared to the control group, and within the SCD genotypes the most elevated levels were found in Hb SS individuals (approximately three-fold higher). Proinflammatory cytokines were higher in SCD groups than in the control individuals. Anemic and hemolytic markers were higher in Hb SS and Hb SD genotypes compared to Hb SC patients. White blood cells and platelets count were higher in Hb SS genotype and were positively correlated to ANXA1 levels. We found that ANXA1 is down-regulated and differentially expressed within the SCD genotypes. Its expression seems to depend on the inflammatory, hemolytic and vaso-occlusive characteristics of the diseased. These data may lead to new biological targets for therapeutic intervention in SCD.

## Introduction

Sickle cell disease (SCD) is a hemolytic anemia caused by the presence of hemoglobin S (Hb S) in homozygous, named sickle cell anemia (SCA), or associated with thalassemias and other hemoglobin variants [1,2]. Phenotypic expression of the SCD is variable and depends on the associated genotype and other factors that alter the hemoglobin concentration or the blood flow. In general, the homozygous inheritance is the most severe form of the disease [3,4]. The beta-globin gene cluster haplotypes associated with Hb S (β^S^-haplotypes) are potential modulators of the phenotypic heterogeneity in SCD, mainly due their relation with fetal hemoglobin (Hb F) levels. There are five typical β^S^-haplotypes: Benin, Bantu, Senegal, Cameroon, and Saudi-Arabian/Indian, of which Bantu usually confers the lowest Hb F levels, causing more severe clinical manifestations [5–7].

The mutation for Hb S occurs in the beta globin gene (*HBB*:c.20A>T; rs334) and it is responsible for the hemoglobin polymerization under conditions of hypoxia, acidosis or dehydration, altering the erythrocytes morphology for a sickling state [1,2]. The association between Hb S and the mutations for hemoglobins C (HBB:c.19G>A; rs33930165) and D-Punjab [(Hb SD); (HBB: c.364G>C)] is also common, and can contribute to the wide phenotypic variety of the disease. As Hb S concentration is a determining factor for the SCD clinical severity, double heterozygous genotypes usually, but not always, are less clinically severe than SCA [3,4]. The Hb SC inheritance is considered a mild genotype [4], while Hb SD results in mild to moderate symptoms, although severe conditions have been reported [8].

The polymerization of Hb S is the primary event in the SCD pathophysiology, resulting in physicochemical changes in erythrocytes and leading to hemolysis and vaso-occlusion [2]. The hemolysis occurs with an early destruction of erythrocytes containing Hb S by releasing hemoglobin and heme iron free in the plasma. Oxidative stress, nitric oxide (NO) depletion, endothelial dysfunction, cell damage, and inflammation are all consequences of this process. Vaso-occlusion is mediated by ischemic-reperfusion cycles, causing tissue damage resulted from oxidative stress, activation of endothelial cells, leucocytes and platelets, increase of adhesion molecules expression, and the release of inflammatory cytokines [9–11].

Inflammation is a key component in SCD pathophysiology [10]. The inflammation response begins when tissue-resident cells of the innate immune system detect the damaging agents and trigger nearby neutrophils. These cells migrate to the inflamated tissue, recruit inflammatory monocytes and potentiate the proinflammatory environment [12,13]. In SCD, the chronic inflammation is characterized by increased leukocytes count and activation of granulocytes, monocytes, and platelets [9,10,14]. Proinflammatory mediators derived from leukocytes, platelets and endothelial cells, such as tumor necrosis factor alpha (TNF-α) and the interleukins (IL), IL-6, IL-1β and IL-8, are usually higher in SCD [15–17]. The increased production and release of proinflammatory cytokines can favor the vaso-occlusive process due to endothelial activation, erythrocytes and leukocytes adhesion to vascular endothelial and endothelial cells apoptosis [15,18].

In order to prevent the progression of inflammation, the inflammatory response must be resolved, promoting the return to homeostasis and inhibiting further tissue damage. The resolution process of the inflammation includes the limitation of neutrophil tissue infiltration, the counter-regulation of cytokines and chemokines, the induction of apoptosis in spent neutrophils and their efferocytosis by macrophages [19]. In this context, annexin A1 (ANXA1) stands out as a potent endogenous anti-inflammatory mediator. ANXA1, also known as lipocortin-1, is a glucocorticoid-regulated protein that is able to reduce neutrophil-endothelial interactions, accelerate neutrophil apoptosis and stimulate macrophage efferocytosis. ANXA1 is expressed mainly in neutrophils, accounting for 2% to 4% of the total intracellular proteins, and its externalization allows an anti-inflammatory action [19–21].

The main mechanism of ANXA1 action is by the inhibition of phospholipase A2 (PLA2), an enzyme involved in the adhesive properties of neutrophils to endothelial cells, preventing the neutrophil transmigration through the endothelium [20,22]. This effect may also be beneficial in ischemia-reperfusion situations [23–25]. Facio et al. (2010) demonstrated that ANXA1 has an important role in renal defense against ischemia-reperfusion injury, aborting neutrophil extravasation after reperfusion [24]. La et al. (2001) and Qin et al. (2014) showed that ANXA1 reduces the tissue damage in the myocardium caused by ischemia-reperfusion events [23,25]. In some chronic inflammatory conditions, as Crohn’s disease or sepsis, ANXA1 levels are usually reduced, supporting the progression and exacerbation of the inflammatory response [26,27]. In SCD, there are no studies about the ANXA1 and its role in the anti-inflammatory response.

SCD is a hemolytic condition, also considered a chronic inflammatory disease, and the neutrophil-endothelium interactions are frequently involved in the vaso-occlusive crisis. Thus, ANXA1 may have an important participation in the SCD pathophysiology. In this study, we evaluate the plasma levels of ANXA1 in SCD patients of three different hemoglobin genotypes, considering a phenotypic graduation from the most to the least severe, according to literature reports: Hb SS, Hb SD and Hb SC, respectively. The results are compared to a control group without hemoglobinopathies (Hb AA). Besides the ANXA1 quantification, we measured plasma levels of proinflammatory cytokines, as well as hematological and biochemical markers as predictors of anemia and hemolysis.

## Results

### Characterization of the study group

Fifty samples from SCD patients were genotyped and we found 24 (48.0%) corresponding to Hb SS, eight (16.0%) to Hb SD and 18 (36.0%) to Hb SC genotypes. All 20 individuals from the control group were confirmed with Hb AA profile (Table 1). We investigated the β^S^ haplotypes and classified them as Bantu and Non-Bantu groups. Non-Bantu group included the other less frequent haplotypes and the atypical combinations. In Hb SS genotype, we found Bantu/Benin (four), Bantu/Atypical (three), Benin/Benin (two) and Benin/Atypical (one) individuals. The atypical haplotypes found in this study were the atypical 1 (- - - - - -) and 3 (- - + - + -), according to classification of Silva et al. (2013) [28]; and one not named haplotype (- + - + - -). In Hb SD and Hb SC, the only non-Bantu haplotype found was Benin. We did not find any differences in the Hb F levels between the two subgroups of haplotypes (Bantu and Non-Bantu) (*Student t-test*; p > 0.05).

**Table 1:** Characterization of the study groups.

Characteristics	Control Group	Hb SS	Hb SD	Hb SC
**Sample size**	20	24	8	18
**Age [years; median (min-max)]**	26.0 (21.0–49.0)	21.0 (12.0–51.0)	20.0 (13.0–43.0)	17.0 (11.0–60.0)
**Gender [n(%)]**				
Female	6 (30.0)	17 (70.8)	3 (37.5)	7 (38.9)
Male	14 (70.0)	7 (29.2)	5 (62.5)	11 (61.1)
**Hb Profile (%; mean ± SD)**				
Hb A	86.3 ± 1.0	NA	NA	NA
Hb A_2_	3.0 ± 0.3	3.4 ± 1.2	2.8 ± 0.4	4.2 ± 0.4
Hb F	0.2 ± 0.3	7.5 ± 5.6	6.3 ± 3.4	1.6 ± 1.4
Hb S	NA	86.0 ± 5.6	43.8 ± 2.8	47.9 ± 0.9
Hb D	NA	NA	42.7 ± 1.7	NA
Hb C	NA	NA	NA	42.3 ± 2.1
**Haplotypes [n (%)]**				
Bantu/Bantu	NA	14 (58.3)	NA	NA
Bantu/Non-Bantu	NA	7 (29.2)	6 (75.0)	16 (88.9)
Non-Bantu/Non-Bantu	NA	3 (12.5)	2 (25.0)	2 (11.1)

Hb: Hemoglobin; SD: standard deviation; NA: not applicable.

### SCD Hemolysis is more severe in Hb SS and Hb SD genotypes

To evaluate the anemic level in our SCD groups, we analyzed hematological parameters within the genotypes (Table 2). Red blood cells (RBC), total hemoglobin and hematocrit were lower in Hb SS and Hb SD individuals compared to Hb SC genotype (p < 0.001). Neutrophil count did not differ between the genotypes, but the total number of white blood cell (WBC) was higher in Hb SS (p < 0.01). Similarly, the platelets count was higher in the homozygous genotype (p < 0.001).

**Table 2:** Hematological profile of the SCD genotypes.

Parameters	Hb SS	Hb SD	Hb SC	P value
RBC (M/μL)	2.7 ± 0.1 ^a^	2.5 ± 0.2 ^a^	4.3 ± 0.2 ^b^	< 0.001
Total Hb (g/dL)	8.2 ± 0.2 ^a^	8.2 ± 0.5 ^a^	11.7 ± 0.4 ^b^	< 0.001
Hematocrit (%)	23.5 ± 0.6 ^a^	22.8 ± 1.6 ^a^	34.1 ± 1.1 ^b^	< 0.001
WBC (K/μL)	11.2 ± 0.6 ^a^	9.3 ± 0.9 ^a,b^	8.1 ± 0.6 ^b^	< 0.01
Neutrophil (K/μL)	5.8 ± 0.5 ^a^	4.3 ± 0.6 ^a^	4.4 ± 0.3 ^a^	0.09
Platelets (K/μL)	534.6 ± 30.3 ^a^	340.0 ± 28.6 ^b^	267.1 ± 29.9 ^b^	< 0.001

RBC: red blood cells. Hb: hemoglobin. WBC: white blood cells. M/μL: million per microliter. g/dL: grams per deciliter. K/μL: thousand per microliter. Statistical analysis: *one-way ANOVA* followed by *Tukey’s post hoc*. Different letters indicate statistical differences.

**Fig 1:**
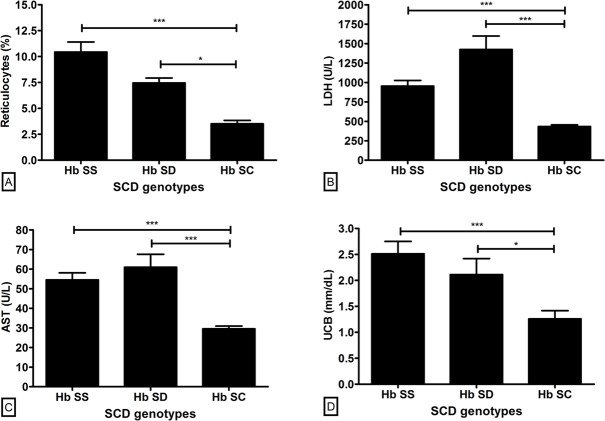
Hemolytic status in the SCD genotypes. (A) Reticulocytes. (B) LDH. (C) AST. (D) UCB. LDH: lactate dehydrogenase. AST: aspartate aminotransferase. UCB: unconjugated bilirubin. U/L: unit per liter. mm/dL: millimeter per deciliter. Reference values: Reticulocytes (1.0–2.6%); LDH (< 480 U/L); AST (< 31 U/L); UCB (< 0.7 mm/dL). Statistical analysis: *Kruskal-Wallis* followed by *Dunn’s* test for A and B; *one-way ANOVA* followed by *Tukey’s* test for C and D. *p < 0.05. ***p < 0.001.

To evaluate the hemolytic profile on patients, we measured the reticulocytes percentage and the circulating values of lactate dehydrogenase (LDH), aspartate aminotransferase (AST) and unconjugated bilirubin (UCB). In all cases, the markers were higher in Hb SS and Hb SD genotypes compared to patients with Hb SC (Fig 1).

### Plasma levels of ANXA1 is decreased in SCD, but higher in Hb SS genotype

**Fig 2:**
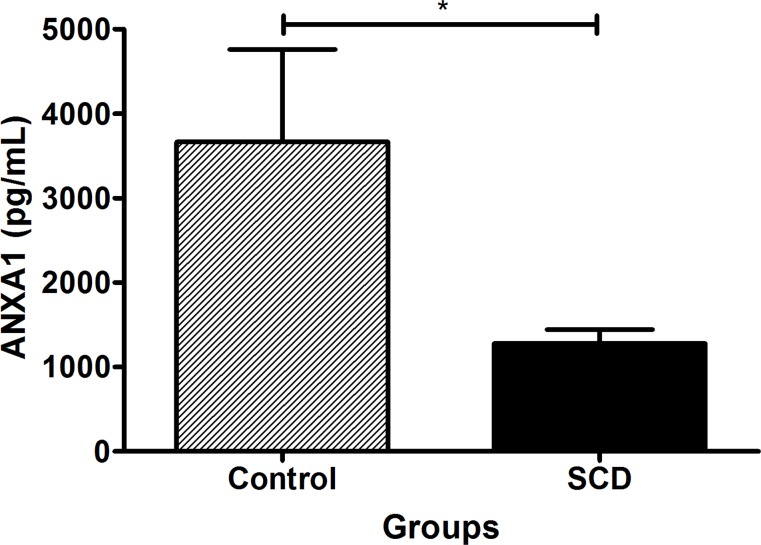
Plasma levels of ANXA1 in control and SCD groups. Data expressed in mean ± standard error of the mean. Statistical analysis: *Student’s t-test*. *p<0.05.

**Fig 3:**
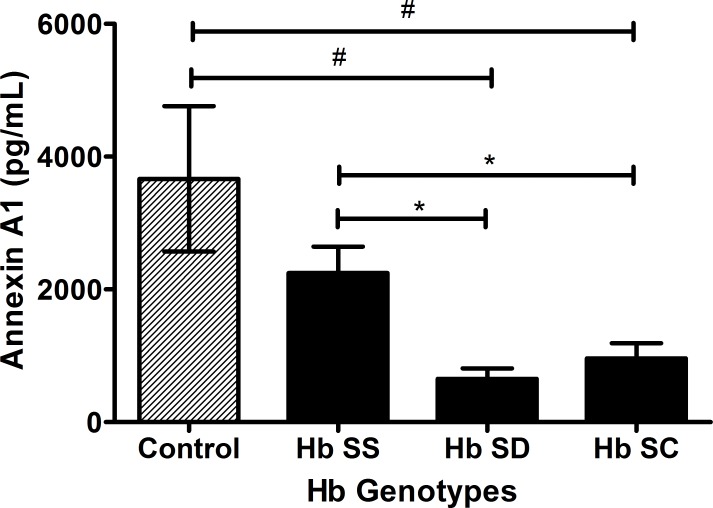
Plasma levels of ANXA1 in control group and SCD genotypes. Data expressed in mean ± standard error of the mean. Statistical analysis: *one-way ANOVA*. *p<0.05 for *Tukey-Krumer post hoc*. ^#^p<0.05 for *Dunnett’s post hoc*.

The plasma levels of ANXA1 were about three-fold lower in SCD patients than in the control group (p = 0.04) (Fig 2). Into SCD group, ANXA1 expression was different between the genotypes (p < 0.01). We found higher plasma levels of ANXA1 in Hb SS than in Hb SD and Hb SC genotypes (p < 0.05), but no difference between Hb SD and Hb SC. Comparing the profile for ANXA1 with the control group and each SCD genotype, we observed statistical differences for Hb SD (p < 0.05) and Hb SC (p < 0.05), but not for Hb SS (Fig 3).

**Fig 4:**
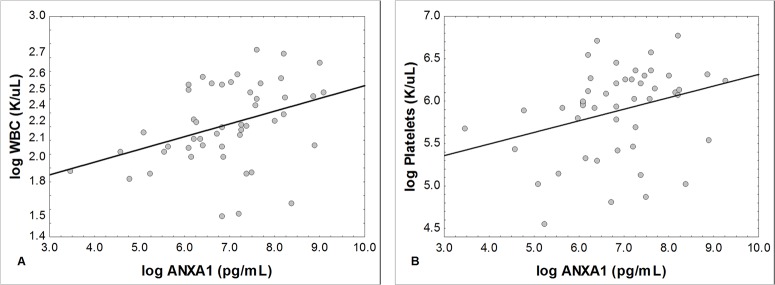
Correlation between plasma levels of ANXA1 and the count of WBC and platelets in SCD patients. (A) ANXA1 *versus* WBC. (B) ANXA1 *versus* Platelets. WBC: white blood cells. Statistical analysis: Pearson’s correlation. Data expressed in logarithm. Moderate correlation between ANXA1 and WBC: r = 0.31; p = 0.03. Moderate correlation between ANXA1 and platelets: r = 0.42; p = 0.01.

Correlation analyses were performed to investigate the relation of ANXA1 with the hemolytic anemia in SCD. The results were not significant between ANXA1 and the hemolytic parameters, but we observed a positive and significant correlation with the WBC (r = 0.31; p = 0.03) and platelets (r = 0.42; p = 0.01) count (Fig 4).

### Proinflammatory state is increased in SCD, especially in Hb SS genotype

**Fig 5:**
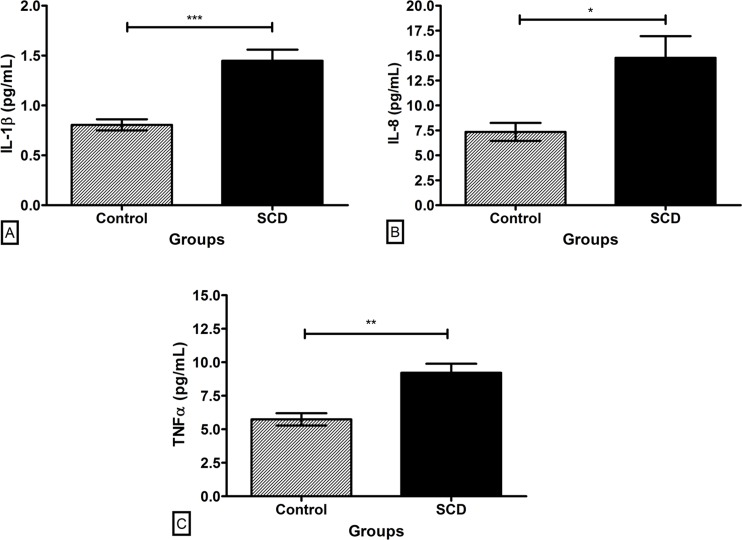
Plasma levels of the proinflammatory cytokines in the control and SCD groups. (A) IL-1β. (B) IL-8. (C) TNF-α. Data expressed in mean ± standard error of the mean. Statistical analysis: Student t-test. *p < 0.05. **p < 0.01. ***p < 0.001.

**Fig 6:**
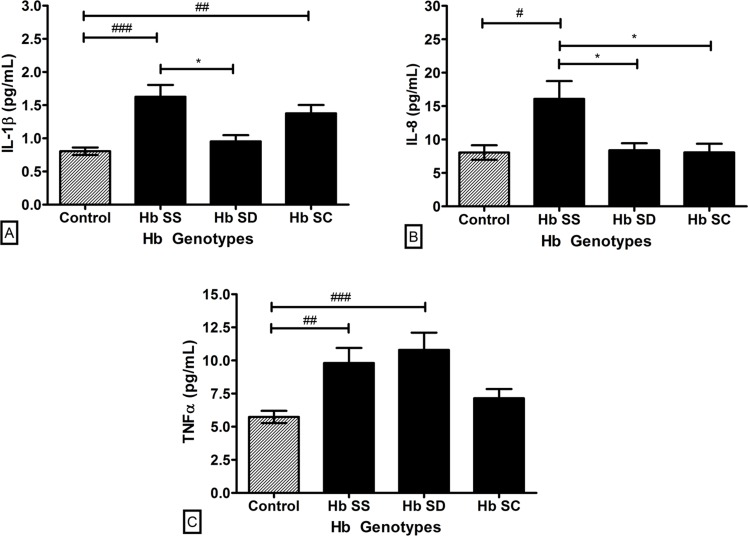
Plasma levels of the proinflammatory cytokines in the control group and SCD genotypes. (A) IL-1β. (B) IL-8. (C) TNF-α. Data expressed in mean ± standard error of the mean. Statistical analysis: *one-way ANOVA*. *p<0.05 for *Tukey-Krumer post hoc*. ^#^*Dunnett’s post hoc* (^#^p<0.05. ^##^p < 0.01. ^###^p < 0.001).

Plasma levels of IL-1β, IL-8, and TNF-α were evaluated in SCD and control groups in order to estimate the inflammatory profile of the samples. All proinflammatory cytokines were increased in SCD patients (Fig 5). Among the SCD genotypes, in general, the highest plasma levels of the cytokines were obtained in the Hb SS genotype (Fig 6). IL-1β plasma levels were different between Hb SS and Hb SD genotypes (p < 0.05). Compared to the control group, the levels were higher in Hb SS and Hb SC (p < 0.001). Regarding IL-8, we observed higher levels in Hb SS compared to double heterozygous genotypes Hb SD and Hb SC (p < 0.05). Homozygous genotype also presented higher levels compared to the control group (p < 0.05). Plasma levels of TNF-α did not exhibit any difference between the SCD genotypes, but the levels in Hb SS and Hb SD were higher than in the control (p < 0.01).

## Discussion

SCD is considered a chronic inflammatory disease due to persistent activation of leukocytes, platelets, and endothelial cells, resulting from hemolysis and vaso-occlusion mediated by ischemia-reperfusion cycles. Most clinical manifestations of the SCD are related to an exacerbation of the inflammatory response and the involvement of cytokines in increasing the hemolytic and vaso-occlusive severity has been evidenced [10,16].

ANXA1 is down-expressed in plasma of SCD patients. ANXA1 is a potent endogenous anti-inflammatory protein expressed in neutrophils and represents a potential therapeutic tool to control inflammatory diseases [29]. ANXA1 regulates neutrophil recruitment to the inflammatory site, induces neutrophil apoptosis, induces monocytes recruitment and promotes efferocytosis. Due to the biological action of ANXA1, low levels of this protein may explain the propagation of the inflammatory response in some chronic inflammatory conditions [30].

This seems to be the first study to evaluate ANXA1 in SCD. Our results corroborate the Sena et al. (2013) and Tsai et al. (2013) findings for Crohn’s disease and sepsis, respectively [26,27]. The authors demonstrated reduced gene and protein expression of ANXA1 in both situations and they suggested a down regulation of ANXA1 as a responsible factor in supporting the inflammatory response.

When subjected to a proinflammatory profile, we observed higher plasma levels of IL-1β, IL-8 and TNF-α in SCD patients compared to control individuals. IL-1β and TNF-α are cytokines that participate in the initial inflammatory response. They are able to promote the production and release of other important mediators, such as IL-8, a neutrophils-attractive chemokine. Together, these and other cytokines act in triggering the inflammatory cascade [31–35]. Our finding demonstrates the inflammatory character of the SCD and suggests some spreading and persistent features of the inflammatory response under low levels of ANXA1.

As expected, among the genotypes, the inflammation was more intense in Hb SS, followed by Hb SD group, except for TNF-α, which did not differ between the SCD groups. For Hb SC, the process was milder, similar to control individuals. Many studies demonstrate that IL-1β production leads to an increase of TNF-α and vice versa. However, in some situations this may not occur [33]. In SCD, a systemic and complex disease, different triggers could favor the beginning of the inflammatory response, explaining the differences between cytokines expression found among the genotypes.

Following these results, the analysis of anemia markers also showed higher severity for Hb SS followed by Hb SD genotype. RBC count, total hemoglobin and hematocrit were lower in these genotypes compared to Hb SC group. Likewise, we investigated the hemolytic profile of the SCD patients by the evaluation of four hemolytic markers as previously validated by Nouraie et al (2013): Reticulocytes percentage, LDH, AST and UCB [36]. All parameters were increased in Hb SS and Hb SD patients, suggesting more severe hemolytic anemia for these genotypes when compared with Hb SC.

The greater severity of the inflammation in Hb SS and Hb SD genotypes is most likely a consequence of the Hb S polymerization and chronic hemolysis. As Hb S concentration is a determinant factor for the SCD clinical severity, double heterozygous genotypes usually, but not always, are less clinically severe than Hb SS [3,37]. However, the similarity found between Hb SS and Hb SD groups may be a result from the Hb D properties to increase the polymerization rate of the Hb S. The mutation responsible for Hb D (*HBB*:c.364G>C) occurs in an interaction site between Hb S molecules and possibly the amino acid change (Glu→Gln) encourages this interaction, promoting the polymerization process and the erythrocyte sickling [38,39].

Despite the inflammatory severity in Hb SS genotype, this group presented the highest levels of ANXA1 among the SCD genotypes. ANXA1 is abundant in neutrophils, which may externalize large amounts of this protein (50% to 70%), especially in response to cytokines released during the inflammatory propagation and neutrophil transmigration [20,22,40]. Homozygous patients for Hb S usually exhibit more anemic, hemolytic, vaso-occlusive and clinical severity than the double heterozygous genotypes [3] and this may represent a potential stimulus for outsourcing and releasing of ANXA1. Moreover, one of the main mechanisms that trigger the ANXA1 externalization is the contact neutrophil-endothelium [20,22], which is a common interaction in the vaso-occlusive processes in SCD due to the cells increased adhesive properties, platelets and endothelium.

Hemolysis, associated to inflammation and large amount of leukocytes and platelets in circulation, could explain the higher plasma levels of ANXA1 found in Hb SS, compared to other genotypes, representing a great stimulus for ANXA1 externalization. According to our data, Hb SD genotype seems to present intermediate characteristics, while Hb SC individuals show a milder phenotype.

Vaso-occlusive episodes are favored by increased adhesive properties of erythrocytes, leukocytes, platelets and endothelial cells in SCD. Besides, a greater number of these circulating factors can aggravate the process [10]. The vaso-occlusion is directly related to inflammatory pathway and involves multiple cell types. Adhesion of platelets or erythrocytes can activate endothelial cells, producing increased expression of adhesion molecules and thus promoting the recruitment of neutrophils. Activate neutrophils roll and adhere to the endothelium, initiating the vaso-occlusive events [14]. We observed that Hb SS individuals presented elevated WBC and platelets count when compared to the other SCD genotypes. In addition, the plasma levels of ANXA1 were correlated to WBC and platelets number, suggesting a link between these blood elements and the release of ANXA1.

We conducted this research on a meticulous selection of study groups based on inclusion and exclusion criteria previously listed by [41]. Furthermore, we considered the possible interference of the β^S^ haplotypes and consequently the Hb F levels in the clinical course of the disease. Our results are consistent and show that ANXA1 protein is down-regulated and differentially expressed between the genotypes in SCD. ANXA1 expression in SCD seems to be dependent on hemolysis severity, inflammatory condition and number of WBC and platelets. Also, ANXA1 is responsible to coordinate the resolution of the inflammation. Despite decreased plasma levels of the ANXA1 in SCD disease, its externalization and releasing can be stimulated by the hemolytic, inflammatory and vaso-occlusive processes. This may represent a compensatory attempt to ease the inflammatory damage in SCD homozygous genotype.

In summary, the results presented indicate that ANXA1 expression is reduced in plasma of SCD patients, but its levels are elevated in homozygous genotype, compared to double heterozygous. These findings suggest that hemolysis and inflammation in Hb SS individuals could be a potential stimuli for ANXA1 externalization and releasing from neutrophils. Our study enlightens the anti-inflammatory role of ANXA1 a promising tool for the development of new therapeutic strategies to treat inflammation in SCD.

## Materials and Methods

### Subjects

The study consisted of 50 SCD patients from the Institute of Hematology *Arthur de Siqueira Cavalcanti* in Rio de Janeiro, RJ, Brazil. All patients were selected according to inclusion criteria, namely: absence of anti-inflammatory prescription for three weeks prior to sample collection, absence of hydroxyurea administration for up to six months preceding the collection date and absence of blood transfusions carried out in <60 days, suggested by Hb A < 10.0% [41]. Only individuals over 10 years old were included in the study, since at this age the hemoglobin profile is usually stable. The control group was composed by twenty volunteers, adults of both genders and with normal hemoglobins, that had not used any anti-inflammatory drugs for the past three weeks. The study has approval by the Research Ethics Committee from Sao Paulo State University (UNESP) under the Certificate of Presentation for Ethics Consideration (CAAE) number 08813112.7.0000.5466. All subjects gave their written consent, which were drawn up in accordance with the ethical guideline regulations for research involving human subjects.

### Samples and genotyping for SCD

Peripheral blood samples (5 mL) were collected into tubes containing 5.0% ethylenediaminetetraacetic acid (EDTA) as anticoagulant. The hemoglobin migration pattern was evaluated by electrophoresis on cellulose acetate at pH 8.6 [42] and agar-agar gel electrophoresis at pH 6.2 [43]. The red cell morphology was analyzed in light microscope with 40x objective lens. The quantification of the hemoglobin fractions was performed by high performance liquid chromatography (HPLC) by VARIANT^TM^ automated equipment (Bio-Rad Laboratories, CA, USA).

In order to confirm SCD genotype by molecular biology, DNA was extracted from leukocytes with phenol-chloroform method [44] and then subjected to polymerase chain reaction followed by restriction fragment analysis (PCR-RFLP). Primers used for amplification of the corresponding region to Hb S and Hb C mutations were 5’-GGCAGAGCCATCTATTGCTTA-3’ and 5’-ACCTTAGGGTTGCCCATAAC-3’. For Hb D-Punjab, the primers were 5’-TGCCTCTTTGCACCATTCTA-3’ and 5’-GA CTCCCACATTCCCTTTT-3’. The amplified segments were treated with specific restrictions enzymes for identification of Hb S, Hb C and Hb D-Punjab mutations: *Dde*I (5’-C↓TNAG-3’), *BseR*I (5’-GAGGAG(N)10/8↓-3’) and *EcoR*I (5’-G↓AATTC-3’), repectively. Fragments obtained were visualized in agarose gel 2.5%.

After genotyping, individuals were separated into three SCD study groups: Hb SS, Hb SD and Hb SC; and the control group: Hb AA.

### Screening of β^S^-haplotypes

DNA samples from SCD patients were submitted to molecular analysis for identification of the β^S^ haplotypes. For this, six polymorphic sites were investigated, namely: 5’γ^G^-*Xmn*I, γ^G^-*Hind*III, γ^A^-*Hind*III, ψβ -*Hinc*II, 3’ψβ-*Hinc*II e 5’β-Hinf I. The technique applied was PCR-RFLP as previously described by Sutton et al. (1989) [45]. The haplotypes were identified by the combination of presence (+) and absence (-) of the respective restriction sites. In this study, one specific haplotype was considered: Bantu, which is defined by the presence of the second restriction site and absence of the other five sites (- + - - - -). Besides being the most frequent β^S^-haplotype in the studied population, Bantu usually confers the lowest levels of Hb F and, consequently, the most severe clinical condition in SCD. Note that Hb SS genotype has two β^S^ clusters, while the other genotypes have only one.

### Hematological and hemolytic parameters in SCD patients

Total hemoglobin, hematocrit and the count of RBC, WBC, neutrophils and platelets were measured by differentiation in flow cytometry and spectrophotometer (Cell-Dyn Ruby). These parameters were used to characterize the study groups and to analyze the intensity of anemia.

Hemolytic profile was evaluated by four markers of hemolysis: reticulocytes percentage, LDH, AST and UCB. These analysis were measured by 2,4- dichlorophenyl diazonio method (Beckman coulter AV680) and performed for a comparison of the hemolytic status between the SCD genotypes.

### ANXA1 and cytokines measurement

The plasma samples were isolated from the total peripheral blood by centrifugation in 500g, for 10 minutes, at 4°C. They were stored at -20°C. The plasma levels of ANXA1 were evaluated by enzyme-linked immunosorbent assay (ELISA). For IL-1β, IL-8 and TNF-α we used multiplex instrument LUMINEX xMAP MAGPIX (Millipore Corporation, Billerica, MA, USA). Technical procedures were performed according to manufacturer’s instructions.

### Statistical analysis

Data were compared between the control group and SCD patients by *Student’s t-*test or *Mann-Whitney* test, depending on the nature of the data (parametric or non-parametric distribution). Comparisons between the SCD genotypes were performed by *one-way ANOVA* or *Kruskal-Wallis* test, followed by *Tukey* or *Dunn post hoc*, respectively. To compare the SCD genotypes against the control group, we applied *one-way ANOVA* followed by *Dunnett’s* test. The correlation analyses were performed by *Pearson* test. In all cases, non-parametric data were transformed in logarithm to prioritize the parametric tests. The adopted confidence interval was 95%, with a significance level of p < 0.05.
