# Correction: Inflammation in Sickle Cell Disease: Differential and Down-Expressed Plasma Levels of Annexin A1 Protein

**DOI:** 10.1371/journal.pone.0172659

**Published:** 2017-02-16

**Authors:** Lidiane S. Torres, Jéssika V. Okumura, Danilo G. H. Silva, Kallyne K. O. Mimura, Édis Belini-Júnior, Renan G. Oliveira, Clarisse L. C. Lobo, Sonia M. Oliani, Claudia R. Bonini-Domingos

There are errors in the Funding section. The correct funding information is as follows: This work was supported by the State of Sao Paulo Research Foudation (FAPESP; grants 2012/21603-2 SMO; 2012/19653-1 LST; and 2016/22239-3 CRBD), by the National Council for Scientific and Technological Development (CNPq; grant 308144/2014-7 SMO), and by the Rio Preto Research Foundation (FAPERP; grant 156/2016 LST), Brazil. The funders had no role in study design, data collection and analysis, decision to publish, or preparation of the manuscript.
